# *Pseudomonas aeruginosa* H3-T6SS Combats H_2_O_2_ Stress by Diminishing the Amount of Intracellular Unincorporated Iron in a Dps-Dependent Manner and Inhibiting the Synthesis of PQS

**DOI:** 10.3390/ijms24021614

**Published:** 2023-01-13

**Authors:** Jinshui Lin, Jianshe Yang, Juanli Cheng, Weipeng Zhang, Xu Yang, Wei Ding, Heng Zhang, Yao Wang, Xihui Shen

**Affiliations:** 1 Shaanxi Key Laboratory of Chinese Jujube, College of Life Sciences, Yan’an University, Yan’an 716000, China; 2 State Key Laboratory of Crop Stress Biology for Arid Areas, Shaanxi Key Laboratory of Agricultural and Environmental Microbiology, College of Life Sciences, Northwest A&F University, Yangling 712100, China; 3 College of Marine Life Sciences, Ocean University of China, Qingdao 266003, China

**Keywords:** *Pseudomonas aeruginosa*, H3-T6SS, H_2_O_2_ stress, Dps, PQS

## Abstract

The type VI secretion system (T6SS), a protein translocation nanomachine, is widely distributed in Gram-negative bacteria and delivers effectors directly into target cells or the extracellular environment to help the bacteria gain a competitive fitness advantage and promote bacterial survival in harmful environments. In this study, we demonstrated that the synthesis of the *Pseudomonas* quinolone signal (PQS) in *Pseudomonas aeruginosa* PAO1 was inhibited by the H3-T6SS gene cluster under iron-rich conditions, and that this inhibition was relieved under iron starvation conditions. Conversely, PQS differentially regulated the expression of the H3-T6SS structural genes and the effector protein gene *tseF*. The expression of *tseF* was inhibited by PQS, while the expressions of the H3-T6SS structural genes were positively regulated by PQS. Further studies showed that the H3-T6SS was involved in the resistance of *P. aeruginosa* to oxidative stress caused by hydrogen peroxide (H_2_O_2_). Interestingly, H3-T6SS expression was neither induced by H_2_O_2_ stress nor regulated by OxyR (a global anti-oxidative transcriptional regulator) but was positively regulated by RpoS (a major transcription regulator of the stress response). In addition, we found that the *clpV3* (a structural gene of H3-T6SS) mutation resulted in upregulation of two proteins related to PQS synthesis and many proteins related to oxidative stress resistance, while the expression of some iron storage proteins, especially Dps, were significantly downregulated. Furthermore, the *clpV3* mutation led to an increase in the intracellular free Fe^2+^ content of *P. aeruginosa*. Further studies showed that both the PQS deficient mutation and overexpression of *dps* effectively restored the H_2_O_2_ sensitive phenotype of the H3-T6SS mutant. Finally, we proposed the following model of H3-T6SS-mediated resistance to H_2_O_2_ stress in *P. aeruginosa*. H3-T6SS not only reduces the intracellular free Fe^2+^ level by upregulating the expression of ferritin Dps, but also inhibits the synthesis of PQS to mediate the resistance of *P. aeruginosa* to H_2_O_2_ stress. This study highlights the important role of H3-T6SS in the ability of *P. aeruginosa* to combat H_2_O_2_ stress and provides a perspective for understanding the stress response mechanism of bacteria.

## 1. Introduction

*Pseudomonas aeruginosa* is a non-fermenting Gram-negative bacterium and is widely distributed in various environments, such as marshes, coastal marine, water, soil, plants and animal tissues due to its strong environmental adaptability [1]. As a common opportunistic pathogen, *P. aeruginosa* causes burn and wound infections as well as chronic respiratory infections in the lungs, especially in cystic fibrosis (CF) patients [1,2]. During host colonization and infection, the pathogen is exposed to stressful environments including oxidative stress [3,4]. Oxidative stress is caused by the Fenton/Haber–Weiss reaction initiated by Fe^2+^ to produce a series of harmful reactive oxygen species (ROS) such as superoxide, hydrogen peroxide and hydroxyl radicals [5]. The production of ROS leads to damage of intracellular macromolecules such as deoxyribonucleic acid (DNA), ribonucleic acid (RNA), proteins and lipids in the cells, resulting in bacterial death or bacteriostasis [5,6]. To survive under oxidative stress, bacteria have developed mechanisms to resist the harmful effects of oxidative stress [5]. Their main strategy is to produce enzymes that degrade ROS such as superoxide dismutase (SOD), catalase and hydroperoxide reductase [3,5].

Recently, accumulated data indicate that the type VI secretion system (T6SS) can also help bacteria to combat oxidative stress [6,7,8]. T6SS, a versatile protein export machine, delivers specialized effectors into neighboring bacterial cells [9], eukaryotic cells [10] or the extracellular environment [11]. Through these effectors, bacterial T6SS is involved in a variety of physiological processes such as biofilm formation [12], metal ion uptake [13,14], bacterial competition [15], antibiotic resistance [16], oxygen limitation response [17] and oxidative stress response [11,13,18]. The antioxidative function of T6SS was first discovered by Weber [19]. Under oxidative stress, the T6SS of *Vibrio anguillarum* is regulated by the general stress response regulator RpoS and is involved in the resistance to H_2_O_2_ stress [19]. Since then, the anti-oxidative stress function of T6SS has been found in a variety of bacteria [8,18,20,21]. For instance, in *Campylobacter jejuni*, the T6SS effector protein TssD plays an important role in anti-oxidative stress [8]. Under oxidative stress, the wild-type (WT) strain exhibits significantly greater resistance to death due to oxidative stress compared to the *tssD* mutant [8]. In addition, the expressions of the *ahpC*, *sodB* and *katA* genes, which encode the proteins involved in the degradation of ROS, are significantly reduced in the *tssD* mutant compared to the WT strain [8]. These results suggest that the *C. jejuni* T6SS is involved in resistance to oxidative stress [8]. Another T6SS effector protein EvpP is essential for the survival of *Edwardsiella piscicida* under oxidative stress [21], suggesting that the T6SS effector protein EvpP facilitates bacterial resistance to oxidative stress [21]. Interestingly, some bacteria resist oxidative stress by ingesting Zn^2+^ and Mn^2+^ [11]. The damage to bacteria caused by oxidative stress can be reduced by acquiring the antioxidant metal ions Zn^2+^ and Mn^2+^, since these two metal ions are important cofactors for enzymes (and their precursors) involved in the degradation of ROS [5]. Under oxidative stress, the expressions of *Yersinia pseudotuberculosis* and *Burkholderia thailandensis* T6SS-4 are induced by OxyR; then, T6SS-4 actively exports metal-binding proteins that facilitate the bacterial acquisition of Zn^2+^ and Mn^2+^ to mitigate potential damage related to oxidative stress [13,22,23]. Together, these results indicate that oxidative stress resistance is a common function of bacterial T6SSs, and different bacterial T6SSs are involved in resistance to oxidative stress via different pathways.

Some bacteria encode multiple sets of T6SSs, which increases the diversity of the T6SSs’ biological functions. For instance, as one of the model bacteria for the study of T6SS, *P. aeruginosa* encodes three T6SSs, namely, H1- to H3-T6SS [24,25]. Among these, the function of H1- and H2-T6SS has been well studied; H1-T6SS mainly mediates bacterial competition [25,26], while H2-T6SS not only mediates bacterial competition but also mediates metal ion uptake and promotes the infection and colonization of *P. aeruginosa* [10,14,17,27,28]. Interestingly, some metal ions, such as molybdate (MoO_4_^2−^) and copper (Cu^2+^), repress the expression of *P. aeruginosa* H2-T6SS in a concentration-dependent manner [14,17]. In addition, the expression of *P. aeruginosa* H2-T6SS is negatively regulated by the iron and ferric uptake regulator (Fur) [29]. Recently, we reported that the expression of *P. aeruginosa* H3-T6SS is also negatively regulated by Fur, and H3-T6SS is involved in the acquisition of the *Pseudomonas* quinolone signal (PQS)-Fe^3+^ [30]. The PQS synthesis cluster has been revealed to be made up of *pqsABCDE*, *pqsH*, *phnAB* and *kynABU* [31,32]. PQS has an iron-chelating activity, and it plays an important role in iron acquisition [30]. In *P. aeruginosa*, PQS chelates Fe^3+^ and forms PQS-Fe^3+^ complexes. TseF (Type VI secretion system effector for Fe uptake) is secreted by H3-T6SS and facilitates the delivery of outer membrane vesicles (OMVs)-associated PQS-Fe^3+^ to bacterial cells by involving the Fe(III)-pyochelin receptor FptA and the porin OprF [30]. This novel iron acquisition pathway highlights the considerable role of H3-T6SS in enhancing bacterial fitness via competition for essential nutrients.

Herein, we reveal that H3-T6SS has a function in mediating the resistance of *P. aeruginosa* to H_2_O_2_ stress. H3-T6SS mainly reduces the content of intracellular free Fe^2+^ by upregulating the expression of the iron storage protein Dps, and it inhibits the synthesis of PQS to combat H_2_O_2_ stress. This finding greatly extends our knowledge of the functional diversity of *P. aeruginosa* T6SS.

## 2. Results

### 2.1. H3-T6SS Inhibits the Synthesis of PQS

**Figure 1:**
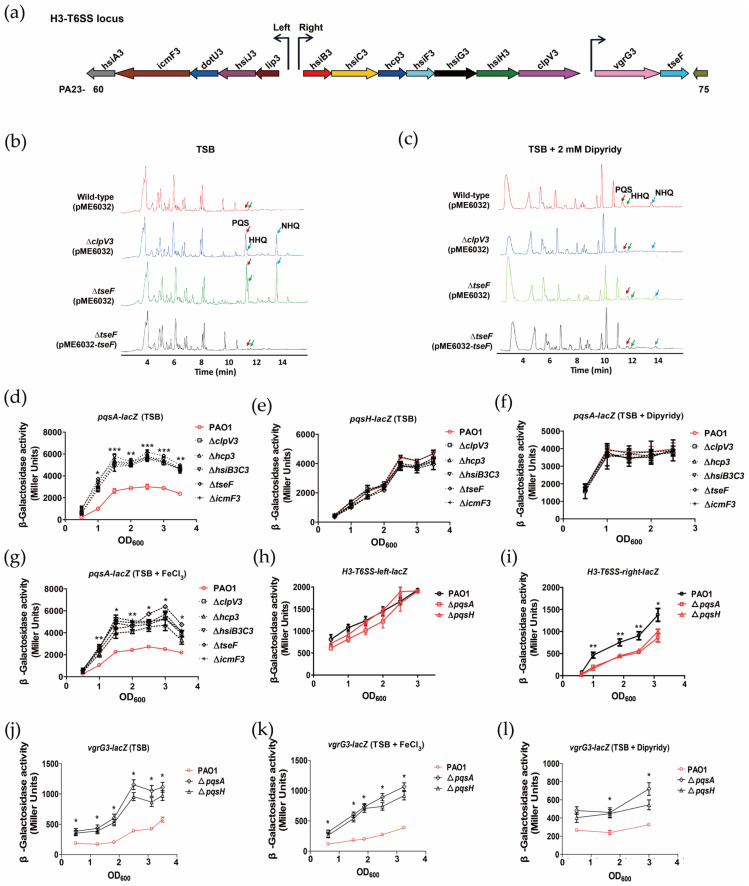
H3-T6SS inhibits the synthesis of PQS, while PQS differentially regulates the expression of the H3-T6SS structural gene and *tseF*. (**a**) H3-T6SS is organized in three putative operons. The genes are labeled *hsiA3* to *hsiJ3* for the left operon, *hsiB3* to *hsiH3* for the right operon, and *vgrG3* and *tseF* for the *vgrG3-tseF* operon. The gene annotation numbers are also indicated (e.g., PA2360). The promoter region of each operon is also shown. (**b**,**c**) LC-MS analysis of cell cultures of relevant *P. aeruginosa* strains. The samples were extracted from the cell culture grown in a TSB medium or in TSB with 2 mM dipyridyl. The mass spectra of PQS, HHQ, and NHQ are shown in Figure S1. (**d**–**g**) Levels of *pqsA* and *pqsH* transcription in *P. aeruginosa* wild-type strain. The H3-T6SS mutants were monitored using *pqsA–lacZ* and *pqsH-lacZ* transcriptional fusion. The experiments were performed in TSB, TSB with 2 mM dipyridyl, and TSB with 200 μM FeCl_3_. (**h**,**i**) Expression patterns of the H3-T6SS left and H3-T6SS right *lacZ* transcriptional fusions for the wild-type strain, Δ*pqsA*, and Δ*pqsH*. The expression is given in Miller units at different OD_600_ during growth at 37 °C in TSB medium. (**j**–**l**) Levels of *vgrG3-tseF* transcription in *P. aeruginosa* WT and PQS-deficient mutants were monitored using the *vgrG3–lacZ* transcriptional fusion. The experiments were performed in TSB, TSB with 200 μM FeCl_3_, and TSB with 2 mM dipyridyl. The graph shows the mean and SD of three experiments performed in five replicates each time. * denotes *p* < 0.05, ** denotes *p* < 0.01, and *** denotes *p* < 0.001 (compared to wild-type).

*P. aeruginosa* H3-T6SS secretes an effector, TseF, which mediates the uptake of PQS-Fe^3+^, and this process is dependent on the outer membrane proteins FptA and OprF [30]. It has been revealed that the *oprF* mutation leads to decreased PQS synthesis in *P. aeruginosa*, while 2-heptyl-4-quinolone (HHQ), a precursor of PQS, is accumulated in cells [33]. To determine whether the synthesis of PQS in *P. aeruginosa* is regulated by H3-T6SS, liquid chromatography-mass spectrometry (LC-MS) was used to detect PQS in *P. aeruginosa* WT PAO1 and H3-T6SS mutant culture medium. In a TSB medium, compared with the WT PAO1, the content of 2-alkyl-4(1H)-quinolone (AQ) derivatives (including PQS, HHQ, and 2-nonyl-4-hydroxyquinoline (NHQ)) in the Δ*clpV3* and Δ*tseF* media increased significantly. Complementation of Δ*tseF* with plasmid pME6032-*tseF* effectively restores cellular AQ derivatives to WT levels (Figure 1b). However, when the bacteria were cultured in a TSB medium containing 2 mM iron chelator 2,2′-dipyridyl, there was no significant difference in the content of AQ derivatives among these strains (Figure 1c). These results indicate that H3-T6SS inhibits the synthesis of PQS in *P. aeruginosa* under the condition of an iron-rich culture; however, the inhibition was relieved under the condition of an iron-deficient culture, suggesting that the regulation of H3-T6SS on the synthesis of PQS depends on the iron content of *P. aeruginosa*.

To further verify that H3-T6SS mutation enhances the synthesis of PQS in *P. aeruginosa*, we constructed chromosomal *lacZ* transcriptional fusion reporter strains of the major PQS synthetic genes *pqsA* and *pqsH* and detected their transcriptional activity in WT PAO1 and H3-T6SS mutants. In the TSB medium, the *pqsA* transcriptional activity of Δ*clpV3*, Δ*hcp3*, Δ*hsiB3C3*, Δ*icmF3* and Δ*tseF* was significantly higher than that of WT PAO1 (Figure 1d), but there was no significant difference in the *pqsH* transcription level among these strains under the same conditions (Figure 1e). These results suggest that the increase in PQS synthesis in *P. aeruginosa* caused by H3-T6SS mutation is mainly achieved by enhancing the expression of the *pqsABCDE* operon. In addition, consistent with the results of the LC-MS analysis, when *P. aeruginosa* was cultured in the TSB medium supplemented with 2 mM 2,2′-dipyridyl, there was no significant difference in the *pqsA* transcription level among these strains (Figure 1f). However, the transcription level of *pqsA* among the mutant strains in the TSB medium supplemented with 200 μM FeCl_3_ was similar to that in the TSB medium, and the level of WT PAO1 was significantly lower than that of the mutants (Figure 1g). These results indicate that the regulation of the *pqsABCDE* operon by H3-T6SS depends on the iron content.

### 2.2. PQS Differentially Regulates the Expression of H3-T6SS Structural Genes and tseF

The H3-T6SS of *P. aeruginosa* PA14 is positively regulated by the signal molecule AQ transcriptional regulator MvfR (multiple virulence factor regulator) [34]. When the synthesis of AQ is inhibited, the expression of H3-T6SS is significantly downregulated [34]. Our data show that although the transcriptional activity of the left gene cluster promoter of H3-T6SS did not change significantly (Figure 1a,h), the transcription level of the right gene cluster promoter of H3-T6SS was significantly reduced in the AQ-deficient mutants Δ*pqsA* and Δ*pqsH* (Figure 1a,i). In addition, we found that Δ*pqsA* and Δ*pqsH* had the same effect on H3-T6SS transcription (Figure 1h,i). However, the *pqsA* mutation makes the cell completely lose the synthesis of the signal molecule AQ, while the *pqsH* mutation only lacks the synthesis of PQS, and the cells can still synthesize HHQ, which is the precursor to PQS [35]. Therefore, the inhibition of the expression of the H3-T6SS major gene cluster in the Δ*pqsA* and Δ*pqsH* mutants is caused by the deletion of PQS, indicating that PQS can activate the expression of the H3-T6SS major gene cluster.

Since PQS can activate the expression of the H3-T6SS major gene cluster, can it also activate the expression of the v*grG3-tseF* operon? The result of the transcriptional fusion of chromosome *vgrG3-lacZ* showed that in TSB, TSB + 200 μM FeCl_3_ and TSB + 2 mM 2,2′-dipyridyl media, the transcriptional activity of *vgrG3-lacZ* was significantly higher in Δ*pqsA* and Δ*pqsH* than in the WT strain (Figure 1a,j–l). This result indicated that PQS negatively regulated the expression of the *vgrG3-tseF* operon and was not affected by the change in the extracellular iron content. The above results suggest that PQS differentially regulates the structural gene of H3-T6SS and *tseF* in *P. aeruginosa* PAO1.

### 2.3. H3-T6SS Plays an Important Role in Combating Oxidative Stress Caused by H_2_O_2_

**Figure 2:**
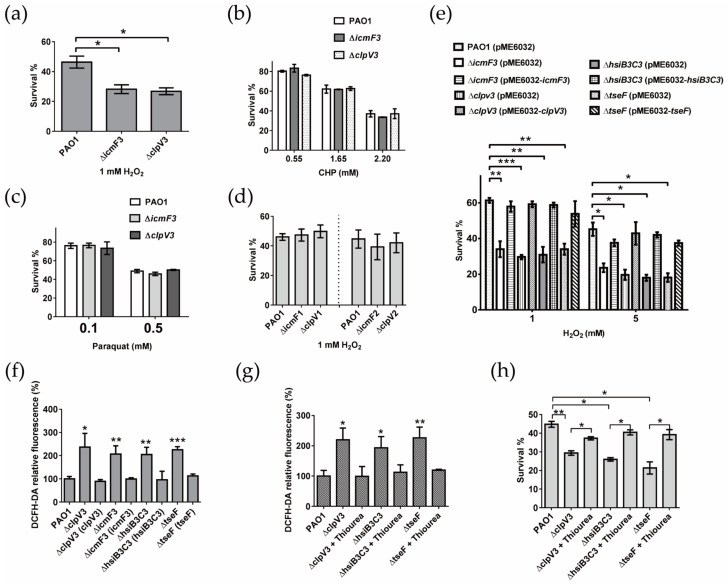
T6SS plays an important role in the H_2_O_2_ stress response. (**a**–**d**) Bacterial strains grown to the stationary phase were exposed to H_2_O_2_, CHP, or paraquat for 1 h, and the viability of the cells was determined. All of these assays were performed in triplicate at least three times. * denotes *p* < 0.05. (**e**) Bacterial strains grown to the stationary phase were exposed to H_2_O_2_ for 1 h, and the viability of the cells was determined. All of these assays were performed in triplicate at least three times. * denotes *p* < 0.05, ** denotes *p* < 0.01, and *** denotes *p* < 0.001. (**f**) Relative intracellular levels of ROS were measured in the stationary-phase planktonic bacteria grown in a TSB medium under H_2_O_2_ stress. The cells were stained with DCFH-DA. The relative levels of fluorescence (excitation = 485 nm; emission = 535 nm) were normalized to the wild-type level under the same conditions. The data for at least three independent experiments were pooled, and the means are shown. The error bars represent the standard deviations. * denotes *p* < 0.05, ** denotes *p* < 0.01, and *** denotes *p* < 0.001 (compared to wild-type). (**g**) Reduction of cellular ROS in the mutants by thiourea. The compound was added to the bacterial cells subjected to H_2_O_2_ stress, and the levels of ROS were measured. (**h**) Thiourea (150 mM) was added to bacterial cells under H_2_O_2_ stress, and their survival rates were determined. The data shown are the average of three independent experiments; and the error bars indicate the SD of the three independent experiments. * denotes *p* < 0.05, and ** denotes *p* < 0.01.

The exogenous addition of PQS makes *P. aeruginosa* more sensitive to H_2_O_2_ and induces the expression of many oxidative stress response genes [36]. Because *P. aeruginosa* H3-T6SS negatively regulates the synthesis of PQS, we hypothesized that H3-T6SS plays a role in the resistance of *P. aeruginosa* to oxidative stress. To this end, the sensitivity differences of *P. aeruginosa* PAO1 and H3-T6SS mutants to the oxidants H_2_O_2_, cumene hydroperoxide (CHP) and paraquat were measured. Our data revealed that mutation of the H3-T6SS structural gene, *icmF3* or *clpV3*, made *P. aeruginosa* more sensitive to H_2_O_2_ (Figure 2a). However, deletion of *icmF3* or *clpV3* did not affect the sensitivity of *P. aeruginosa* to the oxidants CHP and paraquat (Figure 2b,c). These results suggest that the anti-oxidative stress effect of H3-T6SS in *P. aeruginosa* is only specific to H_2_O_2_. However, deleting the structural gene of H1-T6SS or H2-T6SS did not affect the sensitivity of *P. aeruginosa* to H_2_O_2_ under the same conditions (Figure 2d).

Further study revealed that compared with the WT strain PAO1 (pME6032), the survival rate of the mutants Δ*icmF3* (pME6032), Δ*clpV3* (pME6032), Δ*hsiB3C3* (pME6032), and Δ*tseF* (pME6032) decreased significantly under H_2_O_2_ stress, while the H_2_O_2_ stress sensitive phenotypes of these mutants almost completely recovered in complementary strains Δ*icmF3* (pME6032-*icmF3*), Δ*clpV3* (pME6032-*clpV3*), Δ*hsiB3C3* (pME6032-*hsiB3C3*), and Δ*tseF* (pME6032-*tseF*) (Figure 2e). The above results further prove that H3-T6SS plays an important role in combatting H_2_O_2_ stress.

Oxidative stress can induce bacterial cells to produce ROS and destroy proteins, DNA and cell membranes, resulting in apoptosis [5]. Thus, does H3-T6SS exert its anti-H_2_O_2_ stress effect by scavenging ROS? 2′,7′-dichlorofluorescein diacetate (DCFH-DA) is a non-specific fluorescent probe for the detection of the intracellular ROS level in the presence of Fe^2+^ and H_2_O_2_ [37]. Therefore, we used DCFH-DA to detect the intracellular ROS level of H3-T6SS mutants and WT PAO1 under H_2_O_2_ stress. The results revealed that the intracellular ROS levels of the mutants Δ*icmF3* (pME6032), Δ*clpV3* (pME6032), Δ*hsiB3C3* (pME6032), and Δ*tseF* (pME6032) were significantly higher than those of the WT PAO1 (pME6032) strain. After complementation, the intracellular ROS levels of the mutants returned to a level similar to that of the WT PAO1 (pME6032) (Figure 2f). The above results suggest that the antioxidant effect of H3-T6SS under H_2_O_2_ stress is related to its ability to scavenge ROS.

In addition, thiourea, as an effective hydroxyl radical scavenger, can directly quench the hydroxyl radical produced by the Fenton/Haber-Weiss reaction [38,39]. Thiourea treatment could effectively reduce the intracellular ROS level of H3-T6SS mutants under H_2_O_2_ stress (Figure 2g) and could significantly increase the survival rate of H3-T6SS mutants under H_2_O_2_ stress (Figure 2h). These results further confirm that H3-T6SS is involved in the elimination of intracellular ROS in *P. aeruginosa*.

### 2.4. H3-T6SS Is Positively Regulated by RpoS

**Figure 3:**
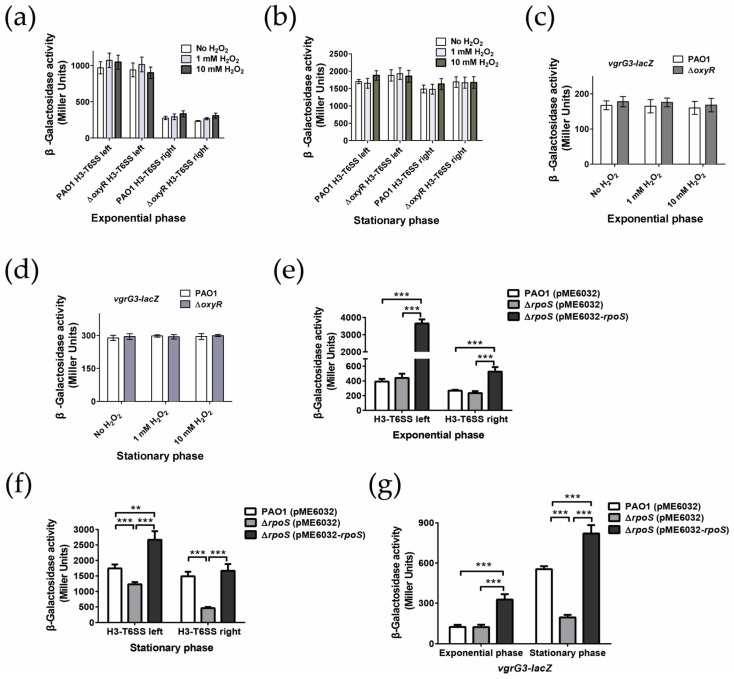
Expression of H3-T6SS is positively regulated by RpoS but not by OxyR. (**a**–**d**) β-Galactosidase analysis of the H3-T6SS promoter activity using the transcriptional *H3-T6SS-left-lacZ*, *H3-T6SS-right-lacZ*, and *vgrG3-lacZ* chromosomal fusion reporters expressed in the wild-type and Δ*oxyR* mutant strains. The H3-T6SS and *vgrG3* expressions were monitored by collecting samples exposed to the indicated concentrations of H_2_O_2_ for 1 h in the logarithmic and stationary growth phases. The graph shows the mean and SD of three experiments performed in five replicates each time. (**e**–**g**) Expression of the *H3-T6SS-lacZ* and *vgrG3-lacZ* transcriptional fusion reporters from the PAO1 strain, its isogenic *rpoS* mutant, and the complementary strain Δ*rpoS* (pME6032-*rpoS*). The growth and β-galactosidase activity were monitored by collecting samples in the logarithmic or stationary growth phase. The graph shows the mean and SD of three experiments performed in five replicates each time. ** denotes *p* < 0.01, and *** denotes *p* < 0.001.

Because H3-T6SS could improve the viability of *P. aeruginosa* under H_2_O_2_ stress, we speculated that the expression of H3-T6SS might be induced by H_2_O_2_. To test this prediction, we constructed chromosome transcriptional fusions of the *P. aeruginosa H3-T6SS-left-lacZ*, *H3-T6SS-right-lacZ*, and *vgrG3-lacZ* reporter strains, and the transcriptional changes in the H3-T6SS major gene cluster and *vgrG3-tseF* in these fusion reporter strains were detected after being subjected to H_2_O_2_ stress for 1 h. Our data revealed that compared with the unstressed WT strain, in both the exponential phase and stationary phase, the promoter activities of the H3-T6SS major gene cluster and *vgrG3* did not change significantly under 1 mM or 10 mM H_2_O_2_ stress (Figure 3a,b). OxyR is a global anti-oxidative transcriptional regulator that senses H_2_O_2_ in *P. aeruginosa* and regulates the expression of many anti-oxidative stress defense genes [40]. The results of the *lacZ* transcriptional fusion enzyme activity revealed that there was no significant difference in the expression level of the H3-T6SS major gene cluster and *vgrG3-tseF* operon between the *oxyR* mutant and WT PAO1, with or without H_2_O_2_ stress (Figure 3c,d). These results suggest that the expression of the entire H3-T6SS gene cluster is not induced by oxidative stress caused by H_2_O_2_ and is not regulated by OxyR.

RpoS, as another major transcription regulator of the stress response in *P. aeruginosa*, regulates the expression of a number of genes in the stationary phase [41]. The transcriptome study revealed that the expression of the H3-T6SS gene cluster was significantly higher in the wild strain of *P. aeruginosa* in the stationary phase than that in the *rpoS* mutant strain [41]. Therefore, we examined whether RpoS regulated the transcription of H3-T6SS via chromosome *lacZ* transcription fusion. In the exponential phase, the transcriptional activities of *H3-T6SS-left-lacZ*, *H3-T6SS-right-lacZ*, and v*grG3-lacZ* were not significantly different in the wild strain PAO1 and the *rpoS* mutant (Figure 3e,g). However, in the stationary phase, the transcriptional activities of *H3-T6SS-left-lacZ*, *H3-T6SS-right-lacZ*, and *vgrG3-lacZ* were significantly lower in the *rpoS* mutant than in the WT strain PAO1 (Figure 3f,g). After complementing pME6032-*rpoS*, the enzyme activity of each promoter in the *rpoS* mutant returned to or even exceeded the level in the WT strain (Figure 3f,g). These results indicate that RpoS positively regulates the expression of the H3-T6SS gene cluster. However, in this study, CTATACT, a conserved binding site of RpoS, could not be identified in each operon promoter region of H3-T6SS, suggesting that RpoS may indirectly regulate the entire H3-T6SS gene cluster.

### 2.5. H3-T6SS Affects the Expression of Various Proteins Related to Oxidative Stress

To further study the H_2_O_2_ stress resistance mechanism of *P. aeruginosa* H3-T6SS, we used quantitative proteomics to compare the difference in protein expression in WT PAO1 and the *clpV3* mutant in the stationary phase. As shown in Table 1, a total of 2365 proteins were detected in the quantitative proteomics, accounting for 42.4% of the entire proteome. Compared with WT PAO1, the expressions of 21 proteins were different after the *clpV3* mutation, accounting for 0.4% of the entire proteome, of which 16 proteins were upregulated and five proteins were downregulated. Compared with the WT strain, the expressions of two proteins related to PQS synthesis, namely PqsD and KynU, were significantly upregulated in the *clpV3* mutant. These results are consistent with the previous results of LC-MS and *pqsA-lacZ* enzyme activity analyses.

The main finding of the quantitative proteomics is that compared with WT PAO1, the upregulated proteins expressed in the *clpV3* mutants are mainly related to oxidative stress, including oxidative stress resistance proteins AhpF, PA3529, and TrxA, molecular chaperones GroEL, GroES, and DnaK in the common stress response, metabolic enzymes AceE and PqqF, which provide reductive power for cells and proteins related to antioxidant defenses, such as PA0102, PA4131, SecB, SecD, and SecF. The downregulated proteins are mainly oxidative stress responsive proteins, such as SdhA, PA1880, and IlvC. These proteins are important metabolic enzymes containing iron-sulfur clusters; they are sensitive to oxidative stress, as iron-sulfur clusters are vulnerable to ROS. These results indicate that H3-T6SS has the function of inhibiting the formation of intracellular ROS. Another major finding of the quantitative proteomics is that compared with WT PAO1, in *clpV3* mutant, the expressions of the two main ferritins BfrA and Dps were downregulated, while the expression of ferredoxin NADP oxidoreductase Fpr was upregulated. However, compared with the WT strain, the expression of BfrA was downregulated 1.6-fold, while the expression of Dps was downregulated 21.8-fold in the *clpV3* mutant. Thus, Dps seems to play a main role in the oxidative stress response. In short, *P. aeruginosa* H3-T6SS differentially regulates the expression of several proteins involved in the oxidative stress response and PQS synthesis.

### 2.6. P. aeruginosa H3-T6SS Combats H_2_O_2_ Stress in Two Ways

The previous results showed that H3-T6SS mutation makes *P. aeruginosa* more sensitive to H_2_O_2_ stress, and H3-T6SS affects the expression of many proteins related to oxidative stress, implying that H3-T6SS mediates the tolerance of *P. aeruginosa* to H_2_O_2_ stress in a variety of ways.

#### 2.6.1. H3-T6SS Combats H_2_O_2_ Stress by Reducing Intracellular Free Fe^2+^

**Figure 4:**
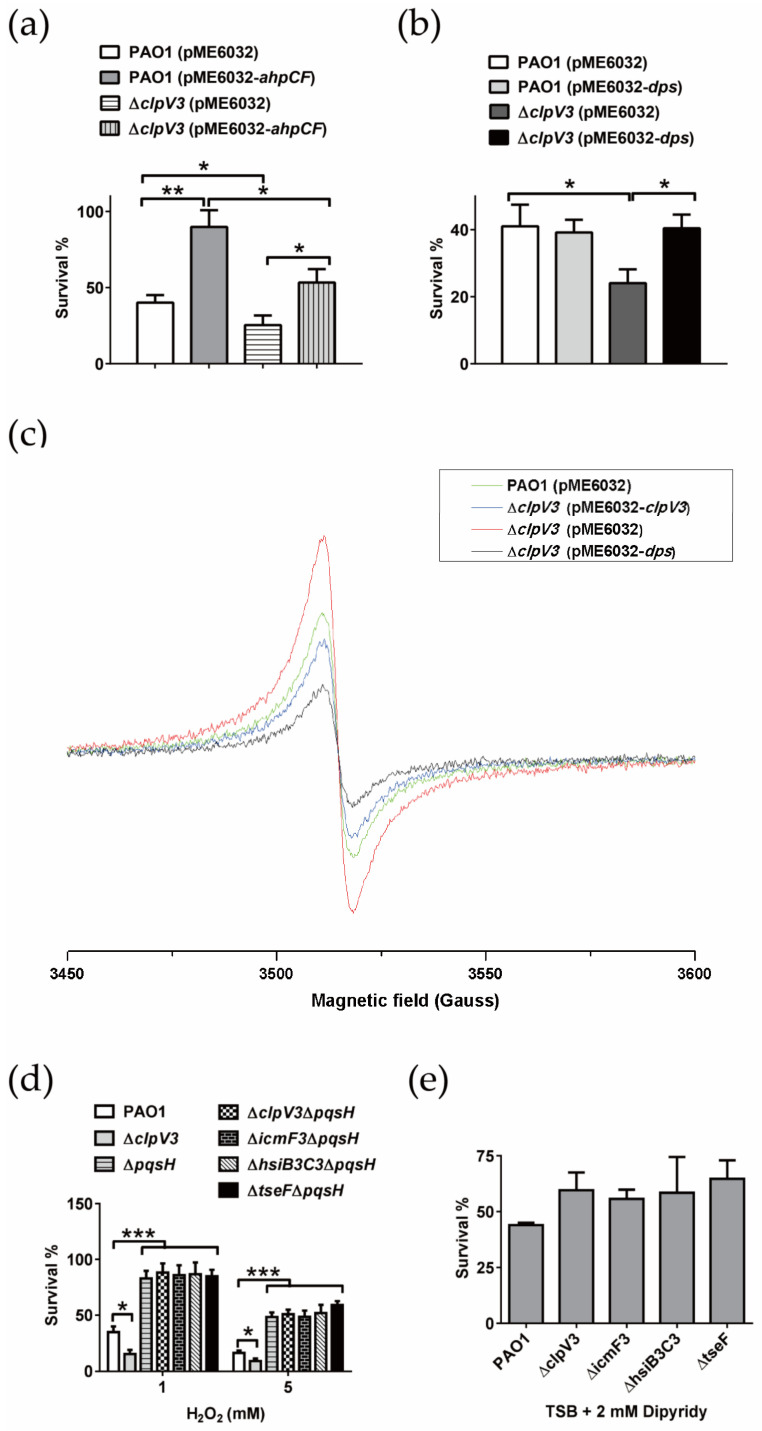
H3-T6SS combats H_2_O_2_ stress in two ways. (**a**,**b**) Survival rates of the *P. aeruginosa* PAO1 (pME6032), Δ*clpV3* (pME6032), PAO1 (pME6032-*ahpCF*), Δ*clpV3* (pME6032-*ahpCF*), PAO1 (pME6032-*dps*) and Δ*clpV3* (pME6032-*dps*) strains were determined after exposure to 1 mM H_2_O_2_ for 1 h. If needed, 1 mM isopropyl-β-d-1-thiogalactopyranoside (IPTG) was included in the medium for induction. The data shown are the average of three independent experiments, and the error bars indicate the SD of three independent experiments. * denotes *p* < 0.05, and ** denotes *p* < 0.01. (**c**) Strains were cultured in TSB medium, and the intracellular free Fe^2+^ content was determined via whole-cell electron paramagnetic resonance (EPR) analysis. *P. aeruginosa* PAO1 (pME6032) is shown in green, Δ*clpV3* (pME6032) is shown in red, Δ*clpV3* (pME6032-*clpV3*) is shown in blue, and Δ*clpV3* (pME6032-*dps*) is shown in black. (**d**) The PQS-deficient mutant reduced the sensitivity of the H3-T6SS mutants to H_2_O_2_. The survival rates of the relevant *P*. *aeruginosa* strains were determined after exposure to 1 mM or 5 mM H_2_O_2_ for 1 h. (**e**) Survival rates of the relevant *P. aeruginosa* strains grown in TSB with 2 mM dipyridyl were determined after exposure to 1 mM H_2_O_2_ for 1 h. The data shown are the average of three independent experiments, and the error bars indicate the SD of three independent experiments. * denotes *p* < 0.05, and *** denotes *p* < 0.001.

Alkyl peroxidase gene cluster *ahpCF* and DNA binding stress protein gene *dps* were overexpressed in the WT PAO1 and Δ*clpV3*, respectively, verifying the proteome results. Under 1 mM H_2_O_2_ stress, the survival rate of Δ*clpV3* (pME6032) was significantly lower than that of strain PAO1 (pME6032). However, the survival rate of the Δ*clpV3* strain after the introduction of pME6032-*ahpCF* was higher than that of the PAO1 (pME6032) strain, but was still significantly lower than that of the PAO1 (pME6032-*ahpCF*) strain (Figure 4a), indicating that overexpression of *ahpCF* could not restore the sensitive phenotype of the Δ*clpV3* strain to H_2_O_2_. Under the same conditions, overexpression of the *dps* gene in the Δ*clpV3* strain could effectively restore the survival rate of the Δ*clpV3* strain to the level of the WT PAO1, which also overexpressed the *dps* gene (Figure 4b). These results suggest that the sensitive phenotype of the Δ*clpV3* strain to H_2_O_2_ was directly related to the decrease in the intracellular Dps protein.

The proteome results showed that the *clpV3* mutation led to a significant downregulation of Dps expression and a slight downregulation of BfrA expression, while the expression of Fpr was significantly upregulated (Table 1). The primary functions of Dps and BfrA are to use O_2_ and H_2_O_2_ as electron acceptors to oxidize intracellular free Fe^2+^ to Fe^3+^ and store Fe^3+^ in the protein via mineralization. The function of Fpr is to reduce the mineralized iron in the intracellular ferritin to free Fe^2+^ and release it [51]. In addition, we found that deleting *clpV3* made *P. aeruginosa* more sensitive to H_2_O_2_, but did not affect the sensitivity of *P. aeruginosa* to CHP and paraquat. The main difference among H_2_O_2_, CHP, and paraquat is that H_2_O_2_ can directly react with Fe^2+^ to produce harmful ROS [5]. Therefore, we speculated that the *clpV3* mutation will lead to an increase in the intracellular free Fe^2+^ content of *P. aeruginosa*. As was expected, the intracellular free Fe^2+^ content of Δ*clpV3* was significantly higher than that of the wild strain PAO1. Complementation of Δ*clpV3* with plasmid pME6032-*clpV3* effectively restored intracellular free Fe^2+^ to WT levels (Figure 4c and Figure S2). Interestingly, overexpression of the *dps* gene also reduced the free Fe^2+^ content of Δ*clpV3* to a lower level than that of WT PAO1 (Figure 4c and Figure S2). These results suggest that the phenotype of elevated intracellular free Fe^2+^ content caused by the *clpV3* mutation is directly related to Dps. The above results confirm the speculation that the sensitivity of H3-T6SS mutants to H_2_O_2_ may be caused by the increase in the mutants’ intracellular free Fe^2+^ content.

#### 2.6.2. H3-T6SS Combats H_2_O_2_ Stress by Inhibiting the Synthesis of PQS

We demonstrated that H3-T6SS mutation led to an increase in PQS synthesis in *P. aeruginosa*. Previous studies have shown that the synthesis of deletion mutant PQS gives *P. aeruginosa* a more resistant phenotype to H_2_O_2_ stress, while the addition or overexpression of the synthetic genes of PQS makes *P. aeruginosa* more sensitive to H_2_O_2_ stress [36,52]. Thus, we may ask, is the phenotype of H3-T6SS mutants that is more sensitive to H_2_O_2_ related to increased PQS synthesis? We compared the sensitivities of different strains to H_2_O_2_ stress. The results are shown in Figure 4d. The survival rate of the Δ*clpV3* mutant was significantly lower than that of wild-strain PAO1, while the Δ*pqsH* mutant had a more resistant phenotype to H_2_O_2_ stress, suggesting that endogenous PQS increased the sensitivity of *P. aeruginosa* to H_2_O_2_ stress. However, under H_2_O_2_ stress, compared with the WT strain, the survival rates of both the Δ*pqsH* and the double mutants of H3-T6SS and *pqsH* significantly increased. The previous results showed that H3-T6SS mutation increased the synthesis of PQS in *P. aeruginosa* in an iron-rich culture and resulted in a more sensitive phenotype to H_2_O_2_ stress. In contrast, under iron-deficient conditions, H3-T6SS mutation did not affect the synthesis of PQS in *P. aeruginosa*. We further tested the sensitivity of *P. aeruginosa* to H_2_O_2_ stress under the same iron-deficient conditions. As shown in Figure 4e, there was no significant difference in the sensitivity to H_2_O_2_ stress between the WT PAO1 and H3-T6SS mutants (Δ*clpV3*, Δ*icmF3*, Δ*hsiB3C3*, and Δ*tseF*) cultured in the TSB medium supplemented with 2 mM 2,2′-dipyridyl. These results suggest that increased PQS synthesis is also one of the reasons that the H3-T6SS mutant is more sensitive to H_2_O_2_ stress.

## 3. Discussion

The role of T6SS in bacterial antagonism has been extensively explored in various environments [25,53,54,55]. However, recent data suggest that this system also plays an important role in combating oxidative stress [18,20,21,56]. For example, KatN, an Mn-containing catalase, could be secreted into the host cell cytosol by enterohaemorrhagic *Escherichia coli* (EHEC) T6SS, thus decreasing the ROS level and enhancing the bacterial survival rate in the host [7]. In *Yersinia pseudotuberculosis*, the expression of T6SS-4 was induced via oxidative stress. The activated T6SS-4 secreted the Zn^2+^-binding effector YezP, which facilitated the acquisition of Zn^2+^ by the bacteria to mitigate potential damage caused by oxidative stress [22]. In this paper, we provide evidence that H3-T6SS primarily reduces the level of intracellular Fe^2+^ by upregulating the expression of Dps, thus mediating the resistance of *P. aeruginosa* to oxidative stress caused by H_2_O_2_. In addition, H3-T6SS also mediates the anti-H_2_O_2_ stress responses of *P. aeruginosa* by inhibiting the biosynthesis of PQS.

**Figure 5:**
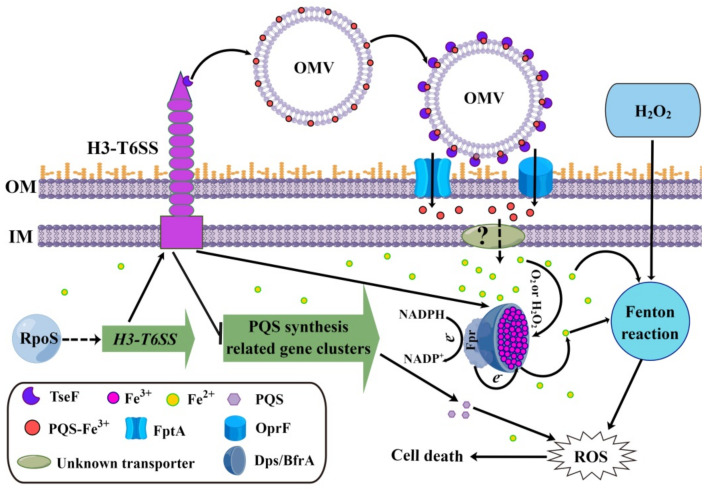
Proposed model of H3-T6SS-mediated resistance to H_2_O_2_ stress in *P. aeruginosa* (created using Figdraw). RpoS indirectly regulates the expression of the H3-T6SS gene cluster. The active H3-T6SS secretes TseF to uptake PQS-Fe^3+^ and depends on OMV, FptA, and OprF [30]. The intracellular free Fe^2+^ undergoes a Fenton reaction with H_2_O_2_ to generate harmful ROS. BfrA and Dps convert intracellular Fe^2+^ into Fe^3+^ and store it as mineralized iron, while Fpr reduces the mineralized iron in the ferritin to free Fe^2+^ and releases it [51]. H3-T6SS mediates the anti-H_2_O_2_ stress of *P. aeruginosa* by upregulating the expression of the iron storage proteins Dps and BfrA and downregulating the expression of the ferredoxin NADP oxidoreductase Fpr, resulting in a reduction of the intracellular free Fe^2+^ content. Furthermore, H3-T6SS inhibits the synthesis of PQS to mediate the anti-H_2_O_2_ stress of *P. aeruginosa*. The dashed arrow represents indirect regulation or unknown mechanism, and the solid arrow represents direct regulation or proven mechanism.

Iron is an essential element for all living organisms. However, iron is toxic when its content exceeds the amount needed for cellular homeostasis, because it can generate ROS via the Fenton reaction [5]. Therefore, tightly controlling the uptake and storage of iron is an important method of regulating intracellular ROS levels and resisting oxidative stress. In many microorganisms, iron is mainly stored in three types of proteins: bacterioferritins, ferritins, and the DNA-binding protein Dps [5]. In addition to providing a source of iron for bacteria under iron-deficient conditions, these three proteins also function to control the ROS generated by the Fenton reaction by sequestering iron ions [5]. For instance, the result of growth inhibition tests on *C. jejuni* revealed that compared to the parental strain, the *dps* mutant was more vulnerable to H_2_O_2_. However, the iron chelator Desferal restored the resistance of the *dps* mutant to H_2_O_2_ [57]. These results suggest that Dps may mainly contribute to protection against oxidative stress by sequestering intracellular free iron to prevent the generation of hydroxyl radicals under H_2_O_2_ stress via the Fenton reaction [57]. Similarly, in this study, we found that compared to the WT strain PAO1, the H3-T6SS mutants were more sensitive to H_2_O_2_ stress (Figure 2a). However, deleting the structural gene of H3-T6SS did not affect the sensitivity of *P. aeruginosa* to the oxidants CHP and paraquat (Figure 2b,c). H_2_O_2_ can directly react with Fe^2+^ to produce harmful ROS, but CHP and paraquat cannot [5], implying that the anti-H_2_O_2_ stress function of *P. aeruginosa* H3-T6SS may be related to the increase in the intracellular free Fe^2+^ content. As was expected, the intracellular free Fe^2+^ content of Δ*clpV3* was significantly higher than that of WT PAO1 (Figure 4c). The intracellular Fe^2+^ level of Δ*clpV3* could be reduced to the level of WT PAO1 by complementing the *dps* gene (Figure 4c). Additionally, the expressions of ferritin BfrA and Dps were positively regulated by H3-T6SS, while the expression of ferredoxin NADP oxidoreductase Fpr was negatively regulated by H3-T6SS (Table 1). Based on the above results, we propose that *P. aeruginosa* H3-T6SS reduces the intracellular free Fe^2+^ content by positively regulating the expression of BfrA and Dps and negatively regulating the expression of Fpr, thus inhibiting the occurrence of the Fenton reaction induced by Fe^2+^ and the formation of intracellular ROS and thereby resisting the H_2_O_2_ stress (see proposed model shown in Figure 5).

PQS, a quorum sensing (QS) signal, differentially regulates *P. aeruginosa* H1-, H2-, and H3-T6SS [34]. Lesic et al. showed that the expression of *vgrG3* in *P. aeruginosa* PA14 was positively regulated by MvfR [34]. However, our results showed that the expression of the *vgrG3-tseF* operon in *P. aeruginosa* PAO1 was negatively regulated by PQS. The differential regulation of PQS on *vgrG3* of *P. aeruginosa* strains PAO1 and PA14 may be due to the different structures of the H3-T6SS gene clusters of these two strains. In the *P. aeruginosa* strain PA14, *vgrG3* and the H3-T6SS major gene cluster are co-transcribed together to form an operon [34]. However, in the *P. aeruginosa* strain PAO1, *vgrG3-tseF* can be transcribed independently. It is worth noting that PQS also sensitizes bacteria toward the bactericidal activity of oxidative stress [32]. A previous study showed that in *P. aeruginosa* PAO1, the *pqs* mutants were more tolerant to H_2_O_2_ than the WT strain, indicating that PQS induces the susceptibility of *P. aeruginosa* to oxidative stress [52]. In this study, we found that compared to the WT strain PAO1, the more sensitive phenotype of the H3-T6SS mutants to H_2_O_2_ was directly related to the synthesis of PQS (Figure 4d). Interestingly, the synthesis of PQS was inhibited by H3-T6SS, indicating that the H3-T6SS mutants were more sensitive to H_2_O_2_, which was directly related to the accumulation of intracellular PQS. Therefore, *P. aeruginosa* H3-T6SS also resists H_2_O_2_ stress by inhibiting the synthesis of PQS (see proposed model shown in Figure 5).

We demonstrated that H3-T6SS mediates the resistance of *P. aeruginosa* to H_2_O_2_ stress by reducing the intracellular free Fe^2+^ content and inhibiting the synthesis of PQS. Thus, we may ask, is H3-T6SS involved in changing the level of intracellular free Fe^2+^ in *P. aeruginosa* and then affecting the synthesis of PQS, or is it involved in affecting the synthesis of PQS and then further regulating intracellular free Fe^2+^ content through PQS? PQS can chelate iron, and a previous study demonstrated that the exogenous addition of 40 μM PQS caused an iron starvation response and significantly inhibited the expression of ferritin BfrB in *P. aeruginosa* PAO1 [36]. In addition, the expression of ferredoxin NADP oxidoreductase Fpr has been found to be inhibited by high concentrations of Fe^3+^ [58], while the expression of ferritins BfrB, BfrA, and Dps is induced by high concentrations of extracellular free Fe^3+^ [59,60,61]. Therefore, we propose that H3-T6SS inhibits the synthesis of PQS, reducing the extracellular PQS, increasing the concentration of extracellular free Fe^3+^ and thus inducing the expression of the iron storage proteins BfrA and Dps. BfrA and Dps convert intracellular Fe^2+^ to Fe^3+^ and store the Fe^3+^ via mineralization, reducing the content of intracellular free Fe^2+^ and thus enhancing the ability of *P. aeruginosa* to resist H_2_O_2_ stress. However, our results revealed that the expression of Dps was not affected by the addition of PQS or the deletion of the *pqsH* gene (data not shown). These results indicate that PQS cannot regulate the expression of Dps, implying that the inhibition of PQS synthesis and upregulation of Dps expression mediated by H3-T6SS independently affect the ability of *P. aeruginosa* to resist H_2_O_2_ stress.

In conclusion, our results revealed the function of H3-T6SS-mediated resistance of *P. aeruginosa* to H_2_O_2_ stress. This function of H3-T6SS mediating H_2_O_2_ stress resistance in *P. aeruginosa* appeared to occur only under iron-rich conditions. However, under iron-limited conditions, *P. aeruginosa* H3-T6SS secretes a PQS-binding effector TseF to recognize and recruit OMVs to the surface of bacterial cells for iron uptake [30]. These results suggest that H3-T6SS plays an important role in the environmental adaptation of *P. aeruginosa* by regulating intracellular iron homeostasis. In addition, these findings greatly expand our current understanding of T6SS-mediated bacterial adaptation to stressful environments and provide a special perspective for understanding the role of T6SS in bacteria–environment interactions. Future studies will focus on the molecular mechanism of the H3-T6SS upregulation of Dps expression and inhibition of PQS synthesis.

## 4. Materials and Methods

### 4.1. Bacterial Strains and Growth Conditions

The bacterial strains and plasmids used in this study are listed in Supplementary Table S1. The *Escherichia coli* strains were grown at 37 °C in either Luria-Bertani (LB) broth or agar. The *P. aeruginosa* strains were grown at 37 °C in either LB, tryptic soy broth (TSB), or succinate minimal medium. The *P. aeruginosa* PAO1 strain was the parent strain of all of the derivatives used in this study. To construct in-frame deletion mutants, the pK18*mobsacB* derivatives were transformed into relevant *P. aeruginosa* strains through *E. coli* S17-1-mediated conjugation and were screened as described by Lin et al. [62]. For overexpression or complementation in the various *P. aeruginosa* strains, the pME6032 derivatives were transformed into the relevant *P. aeruginosa* strains and induced by addition of 1 mM isopropyl-β-D-1-thiogalactopyranoside (IPTG). Antibiotics were used at the following concentrations for *E. coli*: kanamycin (50 μg/mL), gentamicin (15 μg/mL), and tetracycline (15 μg/mL). Antibiotics were used at the following concentrations for *P. aeruginosa*: kanamycin (50 μg/mL), gentamicin (100 μg/mL), chloramphenicol (30 μg/mL), and tetracycline (160 μg/mL for liquid growth or 200 μg/mL for solid growth).

### 4.2. Plasmid Construction

The construction of the knock-out plasmid was modified from a previously reported study [30]. Briefly, to construct the recombinant suicide plasmids for deletion, the 757-bp upstream and 769-bp downstream of the *tseF* (*PA2374*) gene were amplified using a polymerase chain reaction (PCR) with *Pfu* DNA-polymerase, using the primer pairs TseF up F/TseF up R and TseF low F/TseF low R (Supplementary Table S2). The upstream and downstream PCR fragments were ligated using overlap PCR, and the resulting PCR products were inserted into the XbaI/HindIII sites of the suicide vector pK18*mobsacB* to yield the plasmid p-*tseF*. The gentamicin resistance cassette from p34s-Gm was subsequently inserted into the same HindIII site of p-*tseF* to yield the recombinant suicide plasmid pK-*tseF*. The recombinant suicide plasmids pK-*hsiB3C3* (*PA2365-2366*), pK-*clpV3* (*PA2371*), pK-*hcp3* (*PA2367*), pK-*icmF3* (*PA2361*), pK-*icmF1* (*PA0077*), pK-*clpV1* (*PA0090*), pK-*icmF2* (*PA1669*), pK-*clpV2* (*PA1662*), pK-*pqsA* (*PA0096*), pK-*pqsH* (*PA2587*), pK-*oxyR* (*PA5344*), and pK-*rpoS* (*PA3622*) were constructed in a similar manner using primers listed in Supplementary Table S2.

To construct the complementation plasmid pME6032-*tseF*, PCR-amplified *tseF* was cloned into the EcoRI and XhoI sites of plasmid pME6032, producing plasmid pME6032-*tseF*. pME6032-*hsiB3C3*, pME6032-*ahpCF*, pME6032-*clpV3*, pME6032-*icmF3*, pME6032-*rpoS*, and pME6032-*dps* were constructed using the same method.

### 4.3. Construction of Chromosomal Fusion Reporter Strains

The construction of the chromosomal fusion reporter strains were performed using methods with minor modifications that have been previously described [30]. In brief, the *H3-T6SS left*-*lacZ*, *H3-T6SS right*-*lacZ, vgrG3*-*lacZ*, *pqsA*-*lacZ*, and *pqsH*-*lacZ* transcriptional fusions were constructed via PCR amplification of the 508, 508, 1307, 1105, and 1131 bp upstream DNA regions from the *lip3*, *hsiB3*, *vgrG3*, *pqsA*, and *pqsH* genes, using primer pairs PA2364 F/PA2364 R, PA2365 F/PA2365 R, vgrG3 F/vgrG3 R, pqsA F/pqsA R, and pqsH F/pqsH R, respectively (Supplementary Table S2). PCR amplification products from each of the upstream regions were cloned directly into the pMini-CTX::*lacZ* vector [63,64], yielding a range of *lacZ* reporter constructs (see Supplementary Table S1). The promoter fragments were integrated at the CTX phage attachment site (*attB*) in *P. aeruginosa* PAO1, and the relevant mutant strains following established protocols [63,64]. The unmarked transcriptional fusion strains were then constructed via Flp-catalysed excision of the Tc^r^ marker following the established protocols [64].

### 4.4. β-Galactosidase Assays

The β-galactosidase assays were modified from a previously reported study [65]. Overnight bacterial cultures were diluted 1:500 in TSB. If needed, appropriate amounts of oxidants, iron chelators, or FeCl_3_ were included in the medium. The β-galactosidase activity was monitored by collecting samples at different time points. A total of 100 µL of bacterial culture was added to 900 µL of Z Buffer (60 mM Na_2_HPO_4_, 40 mM NaH_2_PO_4_, 10 mM KCl, 1 mM MgSO_4_, pH 7.0, 0.2% β-mercaptoethanol). A total of 1 µL of 0.1% sodium dodecylsulfate (SDS) and 50 µL of chloroform were added to the suspension, which was mixed vigorously for 20 s. The suspension was then incubated for 5 min at 30 °C. A total of 200 µL of 4 mg/mL 2-nitrophenyl β-d-galactopyranoside (ONPG, Sigma) was added to the cells. The reaction was stopped by adding 500 µL of 1 M Na_2_CO_3_. The suspension was centrifuged at 14,000× *g* for 3 min, and the optical densities of the supernatant were read at 420 and 550 nm. The β-galactosidase activity was then calculated in Miller Units (MUs) according to the following equation:

$$\mathrm{MU}=\frac{1000\times \left({\mathrm{OD}}_{420}-1.75\times {\mathrm{OD}}_{550}\right)}{\mathrm{Time}\left(\min\right)\times \mathrm{Volume}\left(\mathrm{mL}\right)\times {\mathrm{OD}}_{600}}$$

### 4.5. Liquid Chromatography-Mass Spectrometry Analysis

The bacteria were cultured in a TSB medium until the optical depth value measured at a wavelength of 600 nm (OD_600_) reached 3.5. The secondary metabolites were extracted using ethyl acetate, which has a moderate polarity and low boiling point. Then, 5 mL of the bacterial culture solution was extracted three times using the same volume of acidified ethyl acetate (containing 0.01% glacial acetic acid), and the extract was dried using a centrifugal concentrator. The extract was analyzed via ultra-performance liquid chromatography (UPLC) (Waters ACQUITY, Milford, MA, USA) using a coupled MicroTOF-MS system (Bruker Daltonics GmbH, Bremen, Germany). A 2.1 mm × 150 mm reversed phase chromatographic column (Waters, BEH C18, 1.7 μm, Milford, MA, USA) with a flow rate of 250 μL min^−1^ was used. The sample was dissolved in acetonitrile and eluted for 30 min using a gradient mobile phase (5% murine, 95% acetonitrile solution containing 1% formic acid). The LC-MS results were processed using DataAnalysis version 3.3 software provided by Bruker Company, Billerica, MA, USA.

### 4.6. Sensitivity Assays

The culture liquid of the TSB overnight culture was transferred to a fresh TSB liquid medium and cultured until the OD_600_ value reached 3.5. One part was diluted in 5 mL of fresh TSB liquid medium and divided into two parts. One part was treated with a certain concentration of oxidant, and the other part was not treated. It was centrifuged at 100 rpm at 37 °C and stressed for 1 h. After the treatment, the cultures were serially diluted and plated onto LB agar plates, and the colonies were counted after 36 h of growth at 37 °C. The survival percentage was calculated by dividing the number of colony-forming units (CFUs) of the stressed cells by the number of CFUs of the unstressed cells. All of these assays were performed in triplicate at least three times.

### 4.7. Intracellular ROS Detection

The intracellular levels of ROS were measured using DCFH-DA as has been previously described by Khakimova et al. [66]. Briefly, the cells were grown in biofilms or to the stationary phase in planktonic cultures. Then, they were washed and resuspended in 1 mL of PBS using the vortex mixing. DCFH-DA (10 μM) was added, and the cells were incubated for 20 min at 37 °C in the dark. The cells were then washed and resuspended in PBS, and the fluorescence was measured at excitation and emission wavelengths of 485 nm and 535 nm, respectively. The fluorescence values were normalized to the OD_600_ value of each sample. To pool the results of the biological replicates, the assay results were calculated as the relative fluorescence normalized to the WT strain.

### 4.8. Proteomic Analysis

The bacteria were cultured in TSB medium until the OD_600_ value reached 3.5. The bacterial cells were collected after centrifugation at 12,000 rpm for 10 min. The supernatant was removed and the pellet was washed twice using a clean medium. The cells were resuspended in SDS protein loading buffer and incubated at 100 °C for 10 min. The proteins were separated using sodium dodecyl-sulfate polyacrylamide gel electrophoresis (SDS-PAGE), and after Coomassie brilliant blue staining, each lane was cut into eight fractions. The protein digestion was performed according to the processes described in a previous study [67]. The gel was cut into small pieces and placed in Eppendorf tubes. Next, 500 μL of 100 mM ammonium bicarbonate/acetonitrile (1:1, *v*/*v*) was added to each tube and the tubes were incubated, with occasional vortexing, for 300 min. Then, 500 μL of acetonitrile was added to the samples, followed by incubation at room temperature for 30 min. The acetonitrile was removed, dithiothreitol solution was added, and the tubes were incubated at 56 °C for 45 min. The dithiothreitol solution was then removed. Next, 55 mM iodoacetamide solution was added, followed by incubation in the dark for 30 min. The gel pieces were shrunk by adding acetonitrile prior to the removal of all of the liquid. Trypsin buffer was added to cover the dry gel pieces, and the sample was incubated at 37 °C for 16 h.

The shotgun proteomics technique was performed using a Thermo Scientific LTQ Velos platform (Thermo Fisher Scientific, Bremen, Germany). The protein digest (1 μg) was separated using a C18 column (Thermo Bio-Basic-18, 150 mm × 0.1 mm, 300 Å pore size, 5 μm particle size) with a flow rate of 150 μL/min. Solvent A was composed of water with 0.1% formic acid, and solvent B was composed of acetonitrile with 0.1% formic acid. Each MS1 was followed by 10 MS2 of the top 10 most abundant ions observed in the MS1 scan.

The acquired raw data were transformed into mgf files using the MM file conversion (v3.9) software. The MS2 spectra were input to Mascot version 2.3.0 (Matrix Science, Ltd., London, UK) to search against translated forward and reverse *P. aeruginosa* PAO1 protein sequences. Decoy sequences consisting of shuffled protein sequences for each of the proteins in the database were also added in the database search. The search criteria were set as follows: tolerances for parent peptides and fragment ions of 1 Da and 0.1 Da, respectively, three missed cleavages, a false discovery rate (FDR) of <1%, carbamidomethylation on cysteins as a fixed modification, and oxidation on methionine, protein N-terminal acetylation and peptide N-terminal pyroglutamate formation as variable modifications. The search results for the two technical replicates of each biological replicate were combined and analyzed together in PeptideProphet. The proteins identified in at least two biological replicates were included in the subsequent analysis. PepC46 was utilized to perform the spectral counting for label-free quantification. It implemented the *t* test and *G* test to analyze the proteomics data based on the MS/MS spectral counts. The technical replicates were combined to increase the spectral counts for each biological replicate. The differentially expressed proteins were filtered using the following cut-off: the *p* value for *t* test was <0.05, the average spectral counts for each protein were at least five, and the fold changes were higher or lower than 1.5-fold. A protein with corrected *p*-values of <0.05 and a fold change of >1.20 or <0.83 was considered to be significantly differentially expressed.

### 4.9. Electron Paramagnetic Resonance Spectroscopy Analysis

The levels of intracellular free iron were measured using a previously reported whole-cell electron paramagnetic resonance (EPR) spectroscopy method [68,69]. Briefly, *P. aeruginosa* strains PAO1 (pME6032), Δ*clpV3* (pME6032), Δ*clpV3* (pME6032-*clpV3*), and Δ*clpV3* (pME6032-*dps*) were cultured in 100 mL of TSB medium. The cells were centrifuged and resuspended in 9 mL of TSB medium, and 1 mL of 0.2 M desferrioxamine mesylate (DF) was added. The resuspended cells were then incubated at 37 °C for 10 min. The cells were centrifuged again, washed with ice-cold 20 mM Tris-HCl (pH = 7.4) buffer, and resuspended in 0.3 mL of Tris-HCl (pH = 7.4) buffer containing 10% (*v*/*v*) glycerol. The samples (0.2 mL) were loaded into 3 mm quartz EPR tubes, and then EPR analysis was performed. The EPR spectrometer settings were the same as in a previous study [69]: a field center of 1570 G, receiver gain of 2500, field sweep of 400 G, modulation amplitude of 1.25 G, temperature of −125 °C, and power of 30 mW.

### 4.10. Statistical Analysis

All of the experiments were performed in triplicate and repeated on two different occasions. The data are expressed as the mean ± S.D. The differences between the frequencies were assessed using the Students *t*-test (bilateral and unpaired), and a *p*-value of 0.05 was considered to be statistically significant. The Shapiro–Wilk test and Levene’s test were performed using the SPSSv13.0 software (SPSS, Chicago, IL, USA) to examine the normality of the data and the homogeneity of the variances, respectively. GraphPad Prism 7, Illustrator (CS6; Adobe, Mountain View, CA, USA), and Figdraw (www.figdraw.com (accessed on 28 December 2022)) were used to create all of the figures.

## Figures and Tables

**Table 1:** Differentially expressed proteins of *P. aeruginosa* PAO1 compared with the *clpV3* mutant identified using quantitative proteomics.

PA No. ^a^	Gene	Fold Change ^b^	*p*-Values	Protein Description ^c^
PQS biosynthesis				
PA0999	*pqsD*	29.6	2.23 × 10^−7^	3-oxoacyl-ACP synthase
PA2080	*kynU*	10.9	7.36 × 10^−3^	Kynureninase KynU
Oxidative processes and oxidative stress				
PA0140	*ahpF*	10.9	7.36 × 10^−3^	Alkyl hydroperoxide reductase
PA3529		10.9	7.36 × 10^−3^	Peroxidase
PA5240	*trxA*	3.4	8.51 × 10^−4^	Thioredoxin
General stress response				
PA4385	*groEL*	15.6	5.92 × 10^−4^	Molecular chaperone GroEL
PA4386	*groES*	29.6	2.23 × 10^−7^	Co-chaperonin GroES
PA4761	*dnaK*	2.3	1.68 × 10^−3^	Molecular chaperone DnaK
PA0962	*dps*	−21.8	8.05 × 10^−7^	DNA-binding stress protein
Genes of the oxidative stress response ^d^				
PA0102		15.6	5.92 × 10^−4^	Carbonic anhydrase
PA5015	*aceE*	3.0	5.12 × 10^−5^	Pyruvate dehydrogenase subunit E1
PA1973	*pqqF*	15.6	5.92 × 10^−4^	Pyrroloquinoline quinone biosynthesis protein F
PA4131		8.6	2.44 × 10^−6^	Iron-sulfur protein
PA3820	*secF*	15.6	5.92 × 10^−4^	Preprotein translocase subunit SecF
PA3821	*secD*	5.1	2.19 × 10^−3^	Preprotein translocase subunit SecD
PA5128	*secB*	10.9	7.36 × 10^−3^	Preprotein translocase subunit SecB
PA1583	*sdhA*	−2.3	4.72 × 10^−2^	Succinate dehydrogenase flavoprotein subunit
PA1880		−2.6	2.32 × 10^−2^	Oxidoreductase
PA4694	*ilvC*	−3.9	7.04 × 10^−6^	Ketol-acid reductoisomerase
Iron sequestration				
PA3397	*fpr*	5.1	2.19 × 10^−3^	Ferredoxin-NADP reductase
PA4235	*bfrA*	−1.6	2.01 × 10^−2^	Bacterioferritin
PA0962	*dps*	−21.8	8.05 × 10^−7^	DNA-binding stress protein

^a^ PA numbers are from http://www.pseudomonas.com (accessed on 10 May 2022). ^b^ Fold changes represent the ratio of the expression levels of *P. aeruginosa* Δ*clpV3* and wild-type PAO1. The minus (−) sign indicates decreased expression in the Δ*clpV3* mutant strain. ^c^ Proteins as described by http://www.pseudomonas.com (accessed on 10 May 2022). ^d^ These genes were identified in transcriptome studies on the response of *P. aeruginosa* to oxidative stress [42,43,44,45,46,47,48,49,50].

## Data Availability

Not applicable.

## Supplementary Materials

The following supporting information can be downloaded at: https://www.mdpi.com/article/10.3390/ijms24021614/s1. References [63,64,70,71,72,73] are cited in supplementary materials.
