# Supplementary material for: Pseudomonas aeruginosa H3-T6SS Combats H2O2 Stress by Diminishing the Amount of Intracellular Unincorporated Iron in a Dps-Dependent Manner and Inhibiting the Synthesis of PQS
Source: Int J Mol Sci. 2023 Jan 13;24(2):1614. doi: 10.3390/ijms24021614 (PMC9866239; doi:10.3390/ijms24021614)
Supplement: Supplementary file 1 [file ijms-24-01614-s001.zip › ijms-2098637-supplementary.pdf]

Supplemental Materials

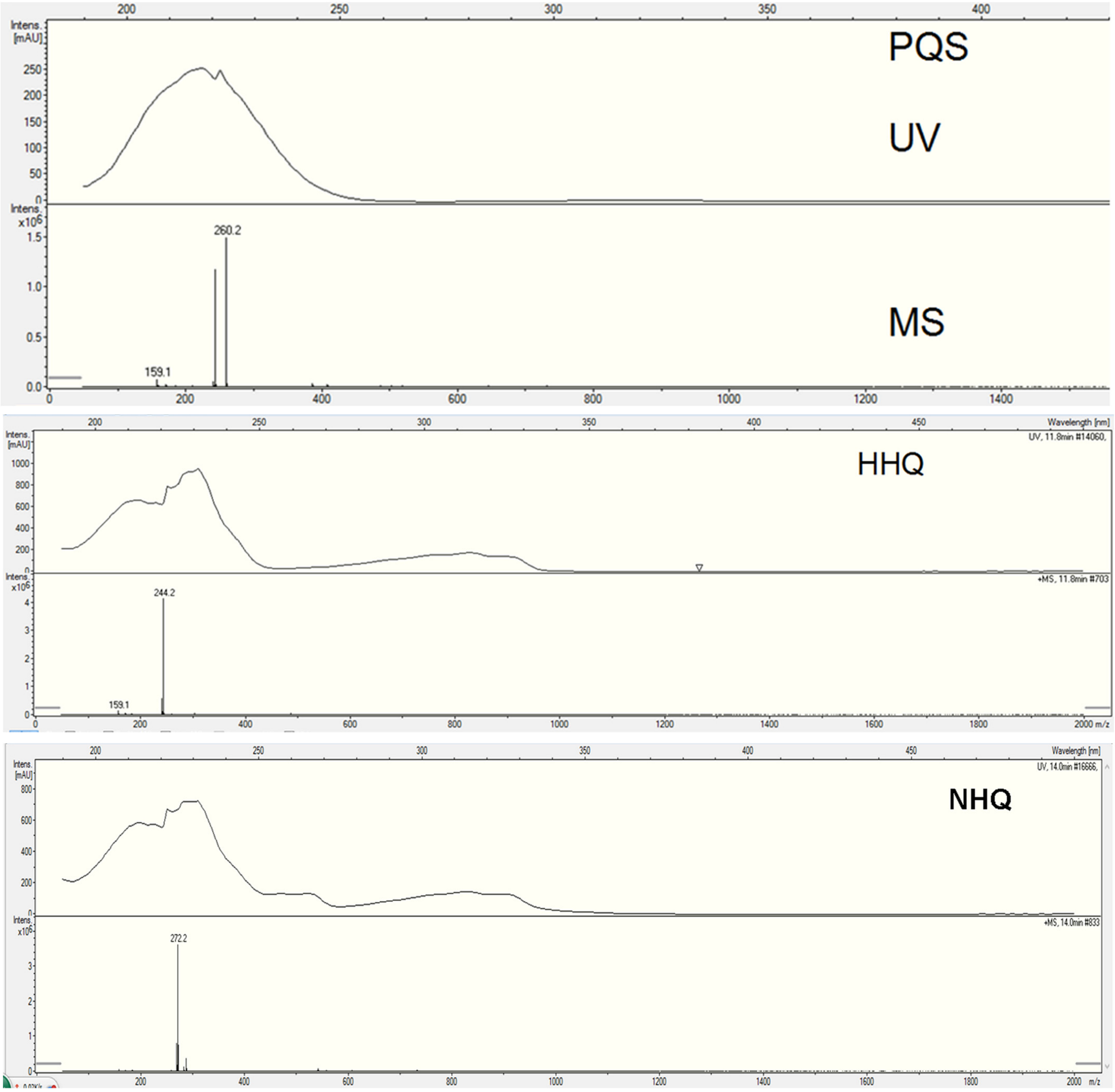

Figure S1. Mass spectra of PQS, HHQ, and NHQ. The bacteria were cultured in TSB medium until the OD_{600} reached 3.5. The secondary metabolites were extracted using ethyl acetate, which has a moderate polarity and low boiling point. Then, 5 mL of the bacterial culture solution was extracted three times using the same volume of acidified ethyl acetate (containing 0.01% glacial acetic acid), and the extract was dried using a centrifugal concentrator. The extract was analyzed via ultra-performance liquid chromatography (UPLC) (Waters ACQUITY, USA) using a coupled MicroTOF-MS system (Bruker Daltonics GmbH, Bremen, Germany). A 2.1 mm × 150 mm reversed phase chromatographic column (Waters, BEH C18,1.7 μm, USA) with a flow rate of 250 μL min^{-1} was used.

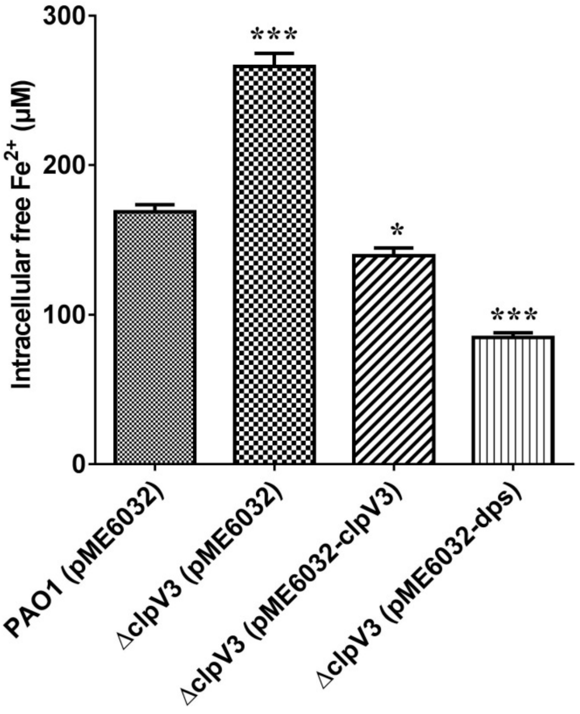

Figure S2. Intracellular free Fe^{2+} concentration of *P. aeruginosa* strains. The strains were cultured in TSB medium, and the intracellular free Fe^{2+} was determined via whole-cell EPR analysis. The data shown are the average of three independent experiments; and the error bars indicate the SD of the three independent experiments. * denotes $P<0.05$, and *** denotes $P<0.001$.

Table S1. Bacterial strains and plasmids used in this study.

Strains and Plasmids	Relevant Characteristics *	Source
Strains		
P. aeruginosa		
PAO1	Wild-type	Laboratory collection
Δ*clpV3*	*clpV3* deletion mutant in PAO1	This study
Δ*hsiB3C3*	*hsiB3*/*hsiC3* double deletion mutant in PAO1	This study
Δ*hcp3*	*hcp3* deletion mutant in PAO1	This study
Δ*icmF3*	*icmF3* deletion mutant in PAO1	This study
Δ*tseF*	*tseF* deletion mutant in PAO1	This study
Δ*icmF1*	*icmF1* deletion mutant in PAO1	This study
Δ*clpV1*	*clpV1* deletion mutant in PAO1	This study
Δ*icmF2*	*icmF2* deletion mutant in PAO1	This study
Δ*clpV2*	*clpV2* deletion mutant in PAO1	This study
Δ*pqsA*	*pqsA* deletion mutant in PAO1	This study
Δ*pqsH*	*pqsH* deletion mutant in PAO1	This study
Δ*oxyR*	*oxyR* deletion mutant in PAO1	This study
Δ*rpoS*	*rpoS* deletion mutant in PAO1	This study
Δ*clpV3*Δ*pqsH*	*clpV3*/*pqsH* double deletion mutant in PAO1	This study
Δ*hsiB3C3*Δ*pqsH*	*hsiB3*/*hsiC3*/*pqsH* triple deletion mutant in PAO1	This study
Δ*icmF3*Δ*pqsH*	*icmF3*/*pqsH* double deletion mutant in PAO1	This study
Δ*tseF*Δ*pqsH*	*tseF*/*pqsH* double deletion mutant in PAO1	This study
PAO1-V	PAO1 with pME6032	This study
PAO1-*ahpCF*	PAO1 with pME6032-*ahpCF*	This study
PAO1-*dps*	PAO1 with pME6032-*dps*	This study
Δ*tseF*-V	Δ*tseF* with pME6032	This study
Δ*tseF*-*tseF*	Δ*tseF* with pME6032-*tseF*	This study
Δ*clpV3*-V	Δ*clpV3* with pME6032	This study
Δ*clpV3*-*clpV3*	Δ*clpV3* with pME6032-*clpV3*	This study
Δ*icmF3*-V	Δ*icmF3* with pME6032	This study
Δ*icmF3*-*icmF3*	Δ*icmF3* with pME6032-*icmF3*	This study
Δ*hsiB3C3*-V	Δ*hsiB3C3* with pME6032	This study

Δ*hsiB3C3*-*hsiB3C3*	Δ*hsiB3C3* with pME6032-*hsiB3C3*	This study
Δ*rpoS* (pME6032)	Δ*rpoS* with pME6032	This study
Δ*rpoS* (pME6032-*rpoS*)	Δ*rpoS* with pME6032-*rpoS*	This study
Δ*clpV3*(pME6032-*ahpCF*)	Δ*clpV3* with pME6032-*ahpCF*	This study
Δ*clpV3* (pME6032-*dps*)	Δ*clpV3* with pME6032-*dps*	This study
E. coli		
DH5*α*	FΦ80Δ*lac*ZΔM15/Δ(*lacZYA*-*argF*)*U169recA1 endA1 hsdR17*	Laboratory collection
S17-1	F$^-$ *thi pro hsdR* [RP4-2 Tc::Mu Km::Tn7 (Tp Sm)]	Laboratory collection
Plasmids		
p34s-Gm	Amp^r; Gm resistant cassette carrying vector	[70]
pK18*mobsacB*	Km^r; *sacB*-based gene replacement vector	[71]
pK-*clpV3*	Km^r, Gm^r; Δ*clpV3*::Gm in pK18*mobsacB*	This study
pK-*hsiB3C3*	Km^r, Gm^r; Δ*hsiB3C3*::Gm in pK18*mobsacB*	This study
pK-*hcp3*	Km^r, Gm^r; Δ*hcp3*::Gm in pK18*mobsacB*	This study
pK-*icmF3*	Km^r, Gm^r; Δ*icmF3*::Gm in pK18*mobsacB*	This study
pK-*tseF*	Km^r, Gm^r; Δ*tseF*::Gm in pK18*mobsacB*	This study
pK-*pqsA*	Km^r, Gm^r; Δ*pqsA*::Gm in pK18*mobsacB*	This study
pK-*pqsH*	Km^r, Gm^r; Δ*pqsH*::Gm in pK18*mobsacB*	This study
pK-*icmF1*	Km^r, Gm^r; Δ*icmF1*::Gm in pK18*mobsacB*	This study
pK-*clpV1*	Km^r, Gm^r; Δ*clpV1*::Gm in pK18*mobsacB*	This study
pK-*icmF2*	Km^r, Gm^r; Δ*icmF2*::Gm in pK18*mobsacB*	This study
pK-*clpV2*	Km^r, Gm^r; Δ*clpV2*::Gm in pK18*mobsacB*	This study
pK-*oxyR*	Km^r, Gm^r; Δ*oxyR*::Gm in pK18*mobsacB*	This study
pK-*rpoS*	Km^r, Gm^r; Δ*rpoS*::Gm in pK18*mobsacB*	This study
pME6032	Shuttle vector between *Pseudomonas* and *E. coli* containing *lacI*^{q}-*Ptac* fragment for gene expression; source of *tetA* gene cassette, Tc^r	[72]
pME6032-*tseF*	*tseF* cloned into pME6032 for complementation	This study
pME6032-*clpV3*	*clpV3* cloned into pME6032 for complementation	This study
pME6032-*icmF3*	*icmF3* cloned into pME6032 for complementation	This study

pME6032-*hsiB3C3*	*hsiB3C3* cloned into pME6032 for complementation	This study
pME6032-*rpoS*	*rpoS* cloned into pME6032 for complementation	This study
pMini-CTX::*lacZ*	Ω-*FRT-attP*-MCS, *ori*, *int*, *oriT*, Tc^r	[63,64]
pFLP2	Amp^r; source of Flp recombinase	[73]
pMP-64	508 bp upstream region of *PA2364* (*H3-T6SS left*) in pMini-CTX::*lacZ*	This study
pMP-65	508 bp upstream region of *PA2365* (*H3-T6SS right*) in pMini-CTX::*lacZ*	This study
pMP-G3	1307 bp upstream region of *vgrG3* in pMini-CTX::*lacZ*	This study
pMP-*pqsA*	1105 bp upstream region of *pqsA* in pMini-CTX::*lacZ*	This study
pMP- *pqsH*	1311 bp upstream region of *pqsH* in pMini-CTX::*lacZ*	This study

*Tc^r, Gm^r, Km^r and Amp^r represent resistance to tetracycline, gentamicin, kanamycin, and ampicillin, respectively.

Table S2. Primers used in this study.

Primiers	5′-3′ Sequence *	
clpV3 up F	CGTGTCTCTAGACCTGTTCCAGCACCGCCTGCTCAG	
clpV3 up R	GCTTCCTCCAGGTCGATGTCCAGCAGCTCCAGCAG	To generate
clpV3 low F	CTGGACATCGACCTGGAGGAAGCCACCCTGGAAGG	pK-V3
clpV3 low R	CACGACAAGCTTGCCGTCGGCAAACTCTTTCGGTAC	
hsiB3C3 up F	TGTCTCTAGATCATACGACTCCCCTTCGACG	
hsiB3C3 up R	CTCGAGCTGGAAGTGCAGCTCCTTCTTCTCGATGGC	To generate
hsiB3C3 low F	GAGCTGCACTTCCAGCTCGAGGAACTCAG	pK-B3C3
hsiB3C3 low R	TGACAAGCTTGAGCCCGTAGGCGAGGATG	
hcp3 up F	CGTGTCTCTAGACACTCCAAGTACCTCTGGGGCAACG	
hcp3 up R	GCGGTCAGCTCCAGGCTGTCGCCTTTGATGTCGCTGC	To generate
hcp3 low F	GGCGACAGCCTGGAGCTGACCGCGCAGAAGGACGAC	pK-P3
hcp3 low R	CACGACAAGCTTCTCGAACTGGACGATCGCGCAGGAC	
icmF3 up F	CGTGTCTCTAGAGATGGTCGAGCGCGCCCTCGAACTG	
icmF3 up R	CAGGACCAGCGGCAGCAGACGCCAGGTGGAGCACAAC	To generate
icmF3 low F	GGCGTCTGCTGCCGCTGGTCCTGCAACTGCCGGTG	pK-F3
icmF3 low R	CACGACAAGCTTCGCGCGCAACGCTTCGTCTAGCTTC	
tseF up F	CTCGTCTAGACGACAACGCCCTGCCCTAC	
tseF up R	CAGCGCCAGCGGTCGCCACGAACCAGGTCGAG	To generate
tseF low F	CGTGGCGACCGCTGGCGCTGGTGGTGGAC	pK-*tseF*
tseF low R	CTCGAAGCTTTGCGGGGAGGCTTCTTGTG	
pqsA up F	CTCGAGATCTCTCGCCCAGTGTACTACG	
pqsA up R	GATAAAGGGTGTCCGAAGGCGAGTCGTTCAAC	To generate
pqsA low F	CTCGCCTTCGGACACCCTTTATCACGACAAC	pK-*pqsA*
pqsA low R	CTCGAAGCTTGCAGCAGTTCATCCAGAC	
pqsH up F	CTCGAGATCTACGGCGAGGTAGTTGTTG	
pqsH up R	CCTCAGCTCGACCAGCAGCCAGTCGATG	To generate
pqsH low F	GCTGCTGGTCGAGCTGAGGAATACCCTCGTTC	pK-*pqsH*
pqsH low R	CTCGAAGCTTGCGAAGACCTGGCGAATC	
icmF1 up F	CGTGTCTCTAGACGCTTCCTCGCCGACGACATCAAGG	
icmF1 up R	TGGTCGAGCAGGATGATCACGTACCAGGGCAACTCGTAC	To generate
icmF1 low F	GTACGTGATCATCCTGCTCGACCAGTCGGACCTGGAAC	pK-F1
icmF1 low R	CACGACAAGCTTGCCGGGAAAGAGCGGGATCACCTC	

clpV1 up F	GTGCGAATTCACCGCACGCTACTACTACACCG	
clpV1 up R	CAGCGCAACGCGACTGAT	To generate
clpV1 low F	ATCAGTCGCGTTGCGCTGGACGCGATCCTCACCAACA	pK-V1
clpV1 low R	GTGCAAGCTTGGCGGAACCAGTAGTAGATGC	
icmF2 up F	CGTGTCTCTAGACGAACAGCTCGAGGAGCGGATGATC	
icmF2 up R	GAGCACCATCACCAGGAGTACCAGCAACAGCGTCCAG	To generate
icmF2 low F	CTGGTACTCCTGGTGATGGTGCTCAAGGCCGACCTG	pK-F2
icmF2 low R	CACGACAAGCTTCGGTTCGTTCATTGGCGGATCACCAC	
clpV2 up F	CGTGTCTCTAGAACCTGATCGGCTTGTTCGGTGCCAG	
clpV2 up R	CAGCAAACGGTCGGTGTCGGCATCCAGCGCCTGAATC	To generate
clpV2 low F	GATGCCGACACCGACCGTTTGCTGGAAGCGATGGC	pK-V2
clpV2 low R	CACGACAAGCTTGCAGTTCTGCACCACGAACGCCTG	
oxyR up F	CGTGTCTCTAGATGCTCGGCGTCGAGCATGATCCGTC	
oxyR up R	CGGAACGGCACGACCGAGAGGGTCGGTTGGCTGAC	To generate
oxyR low F	GACCCTCTCGGTCGTGCCGTTCCGTACCGTTGCCATCG	pK-*oxyR*
oxyR low R	CACGACAAGCTTTGGCCAGGGCCTGTTGGCTCAACTG	
rpoS up F	CGAGCAGGATCCTATCCAATTTGGTGGGCGGGCATC	To generate
rpoS up R	GACCTCTTCCAGCTGCTCGTCGGCAGACGACTCG	pK-*rpoS*
rpoS low F	GCCGACGAGCAGCTGGAAGAGGTCGGCCAGGAAATC	
rpoS low R	GCTCGAGCATGCATCCATCCGGACGAGTGCATCGAC	
tseF F	CTCGGAATTCATGGCGGCATCCGGCAAG	To generate
tseF R	CTCGCTCGAGGATGCGCGCCTAGGGCTC	pME6032-*tseF*
clpV3 F	CTCGGAGCTCCTGCGGCAGCCGGAGGTAG	To generate
clpV3 R	CTCGAGATCTGTCCTACTCCAACACCCACTCC	pME6032-*clpV3*
icmF3 F	CTCGGAATTCATGAGCGGCGCGACGCTG	To generate
icmF3 R	CTCGAGATCTCATGGGGTACCTCCGGTGCTG	pME6032-*icmF3*
hsiB3C3 F	CTCGGAGCTCTCCCCCACCATTGCATCG	To generate
hsiB3C3 R	CTCGGGTACCGAGGTGCGCTCCGAGTTG	pME6032-*hsiB3C3*
rpoS F	CTCGGAATTCTGAGTCGAACTCATGCAAGGG	To generate
rpoS R	CTCGAGATCTTTCCGTCACTGGAACAGCG	pME6032-*rpoS*
aphCF F	CTCGGAGCTCCCCATTCAACTGAGAGAGGAAC	To generate
aphCF R	CTCGGGATCCGCTGGCCGGGTCACTCCG	pME6032-*aphCF*
dps F	CTCGGAATTCATGGAAATCAATATCGGAATCGG	To generate
dps R	CTCGAGATCTCGATCGACGATCAGCTGG	pME6032-*dps*

PA2364 F	TGTCGGTACCGAAGCGCAGCTCGACGTTC	To generate
PA2364 R	TGTCGAATTCCGGGACCAGCTCCAGGCTC	pMP-64
PA2365 F	TGTCGGTACCCGGGACCAGCTCCAGGCTC	To generate
PA2365 R	TGTCGAATTCGAAGCGCAGCTCGACGTTC	pMP-65
vgrG3 F	CCTGCTCGAGATCCTCGCCACCAGCAAC	To generate
vgrG3 R	TGTCCTGCAGGGTGGGACGGGGCATTAGTG	pMP-G3
pqsA F	CTCGGTCGACAACCCACCGGCGAAACCG	To generate
pqsA R	CTCGCTGCAGGCATTGCAGCCGGCTGAG	pMP-*pqsA*
pqsH F	CTCGCTCGAGGGAGGGTGAAGGTGATGCC	To generate
pqsH R	CTCGAAGCTTGGTACCGATGGGCCTGATC	pMP- *pqsH*

*Underlined sites indicate restriction enzyme cutting sites added for cloning.

Supplementary References

63. Becher, A.; Schweizer, H.P. Integration-proficient *Pseudomonas aeruginosa* vectors for isolation of single-copy chromosomal *lacZ* and *lux* gene fusions. *Biotechniques.* **2000**, 29, 948-950, 952.
64. Hoang, T.T.; Kutchma, A.J.; Becher, A.; Schweizer, H.P. Integration-proficient plasmids for *Pseudomonas aeruginosa*: site-specific integration and use for engineering of reporter and expression strains. *Plasmid.* **2000**, 43, 59-72.
70. Dennis, J.J.; Zylstra, G.J. Plasposons: modular self-cloning minitransposon derivatives for rapid genetic analysis of gram-negative bacterial genomes. *Appl Environ Microbiol.* **1998**, 64, 2710-2715.
71. Schafer, A.; Tauch, A.; Jager, W.; Kalinowski, J.; Thierbach, G.; Puhler, A. Small mobilizable multi-purpose cloning vectors derived from the *Escherichia coli* plasmids pK18 and pK19: selection of defined deletions in the chromosome of *Corynebacterium glutamicum*. *Gene.* **1994**, 145, 69-73.
72. Heeb, S.; Blumer, C.; Haas, D. Regulatory RNA as mediator in GacA/RsmA-dependent global control of exoproduct formation in *Pseudomonas* fluorescens CHA0. *J Bacteriol.* **2002**, 184, 1046-1056.
73. Hoang, T.T.; Karkhoff-Schweizer, R.R.; Kutchma, A.J.; Schweizer, H.P. A broad-host-range Flp-FRT recombination system for site-specific excision of chromosomally-located DNA sequences: application for isolation of unmarked *Pseudomonas aeruginosa* mutants. *Gene.* **1998**, 212, 77-86.
